# Distinct transcriptome profiles of Gag-specific CD8+ T cells temporally correlated with the protection elicited by SIVΔnef live attenuated vaccine

**DOI:** 10.1371/journal.pone.0173929

**Published:** 2017-03-23

**Authors:** Wuxun Lu, Yanmin Wan, Fangrui Ma, R. Paul Johnson, Qingsheng Li

**Affiliations:** 1 School of Biological Sciences, University of Nebraska-Lincoln, Lincoln, Nebraska, United States of America; 2 Nebraska Center for Virology, University of Nebraska-Lincoln, Lincoln, Nebraska, United States of America; 3 New England Primate Research Center, Harvard Medical School, Boston, Massachusetts, United States of America; University of Pittsburgh Centre for Vaccine Research, UNITED STATES

## Abstract

The live attenuated vaccine (LAV) SIVmac239Δnef (SIVΔnef) confers the best protection among all the vaccine modalities tested in rhesus macaque model of HIV-1 infection. This vaccine has a unique feature of time-dependent protection: macaques are not protected at 3–5 weeks post vaccination (WPV), whereas immune protection emerges between 15 and 20 WPV. Although the exact mechanisms of the time-dependent protection remain incompletely understood, studies suggested that both cellular and humoral immunities contribute to this time-dependent protection. To further elucidate the mechanisms of protection induced by SIVΔnef, we longitudinally compared the global gene expression profiles of SIV Gag-CM9+ CD8+ (Gag-specific CD8+) T cells from peripheral blood of Mamu-A*01+ rhesus macaques at 3 and 20 WPV using rhesus microarray. We found that gene expression profiles of Gag-specific CD8+ T cells at 20 WPV are qualitatively different from those at 3 WPV. At 20 WPV, the most significant transcriptional changes of Gag-specific CD8+ T cells were genes involved in TCR signaling, differentiation and maturation toward central memory cells, with increased expression of CCR7, TCRα, TCRβ, CD28 and decreased expression of CTLA-4, IFN-γ, RANTES, granzyme A and B. Our study suggests that a higher quality of SIV-specific CD8+ T cells elicited by SIVΔnef over time contributes to the maturation of time-dependent protection.

## Introduction

A safe and effective prophylactic vaccine is an ultimate solution to human immunodeficiency virus type 1 (HIV-1) pandemic; however, it remains elusive after 3 decades of extensive research. Among all the vaccine modalities tested in rhesus macaque/SIV model for HIV-1 vaccine study, SIVmac239 with *nef* gene deletion (SIVΔnef), a live attenuated vaccine (LAV), induces the most potent protection against pathogenic SIV challenges via intravenous or mucosal routes [1, 2]. It achieved 93% (59/63) protection in vaccinated macaques [3]. Despite the potent protection induced by SIVΔnef LAV, it was revealed that the pathogenicity in neonatal macaques after infection with SIVΔ3, a LAV with deletion in *nef*, *vpr* and LTR regions [4], manifested with high viremia and AIDS development. A prolonged follow-up study in adult macaques also showed that most macaques vaccinated with SIVΔ3 LAV had immune dysregulation, and 18% (2/11) developed AIDS [5]. Although the potential risks of inducing immune dysregulation and even AIDS preclude HIV-1 LAV for human use, a better understanding of the underlying mechanisms of potent protection induced by SIVΔnef LAV may facilitate development of safe HIV-1 vaccines with improved efficacy.

The protection induced by SIVΔnef LAV shows a unique time-dependent pattern. SIVΔnef replicates efficiently in rhesus macaques after vaccination. Plasma viral load peaks at 7–12 days post-inoculation, but drastically declines to undetectable levels at 5 weeks post-vaccination (WPV) [6]. There is no or very limited protection against intravenous challenge with pathogenic wild-type SIVmac251 at 5 WPV, but potent protection arises at 15 WPV and thereafter [6]. The prolonged delay of emergence of protection against subsequent SIV challenge after SIVΔnef LAV indicates there is an immune maturation over time [6–8]. It has been shown that the time-dependent protection induced by SIVΔnef LAV is associated with vigorous SIV-specific CD8+ T cell responses [2, 9–12], but not neutralizing antibodies [2, 13]. In our recent studies, we found that IgG antibodies specific to SIV gp41 trimers with limited neutralizing activities correlated spatially and temporally with the maturation of local protection against high-dose pathogenic SIV vaginal challenge [14], but SIV-specific CD8+ T cells quantitatively did not correlate with maturation of vaginal protection [15, 16]. However, after SIVΔnef vaccination, the transcription factor profiles of SIV-specific CD8+ T cells in peripheral blood changed over time and temporally associated with the protection, indicating SIV-specific CD8+ T cells elicited by SIVΔnef are qualitatively different between time points of un-protection and protection [17].

To further elucidate the mechanisms of protection induced by SIVΔnef vaccine, in this study, we longitudinally compared the global gene expression profiles of SIV Gag-specific CD8+ T cells targeting a dominant protective epitope CM9, which is restricted by the Mamu-A*01 MHC class I allele [18, 19], from peripheral blood of rhesus macaques at 3 and 20 WPV using rhesus microarray. We found that gene expression profiles of Gag-specific CD8+ T cells at 20 WPV are qualitatively different from those at 3 WPV. At 20 WPV, the most significant transcriptional changes of Gag-specific CD8+ T cells were genes involved in cell TCR-signaling, T cell differentiation and maturation toward central memory cells. Our study indicates that a higher quality of SIV-specific CD8+ T cells elicited by SIVΔnef LAV over time contributes to the maturation of time-dependent protection.

## Materials and methods

### Ethics statement

Five adult female rhesus macaques (*Macaca mulatta*) of *Indian* origin were used in this longitudinal study. and the macaques were housed in New England Primate Research Center (NEPRC) in accordance with the regulations of the American Association of Accreditation of Laboratory Animal Care and standards of the Association for Assessment and Accreditation of Laboratory Animal Care International (AAALAC) as described previously [17]. The experiments and procedures of this study were approved by Institutional Animal Care and Use Committee at Harvard Medical School (protocol 04383). All animals were housed in an AAALAC-accredited facility, and husbandry and care met the guidance of Animal Welfare Regulations and standards in The Guide for the Care and Use of Laboratory Animals. All animals were enrolled in the NEPRC behavioral management program, including an IACUC-approved plan for Environmental Enrichment for research primates. Enrichment was provided through manipulable devices, foraging opportunities, food items, structural and environmental enhancements, and positive human interaction. Enrichment devices were rotated on a weekly basis and included toys, mirrors, radios, TV/VCRs, foraging boards, and a variety of complex foraging devices.

This protocol had an IACUC-approved exemption from social housing based on scientific justification. Primary enclosures consisted of stainless steel primate caging provided by a commercial vendor. Animal body weights and cage dimensions were regularly monitored. Overall dimensions of primary enclosures (floor area and height) met the specifications of The Guide for the Care and Use of Laboratory Animals, and the Animal Welfare Regulations (AWR). Further, all primary enclosures were sanitized every 14 days at a minimum, in compliance with AWRs. Secondary enclosures (room level) met specifications of The Guide with respect to temperature, humidity, lighting and noise level. The animals were provided ad lib access to municipal source water, offered commercial monkey chow twice daily, and offered fresh produce a minimum of three times weekly. Light cycle was controlled at 12/12 hours daily. The animals were subject to twice daily including weekend documented observations by trained animal care and veterinary staff. If signs of illness or distress were noted the veterinarians were immediately notified. The animals were examined and supportive therapy given as required. There were not any of the animals become severely ill or died at any time prior to the experimental endpoint. To alleviate the non-human primates' suffering, all invasive procedures were performed after anesthesia. Euthanasia took place for all the animals at endpoints using protocols consistent with the American Veterinary Medical Association (AVMA) guidelines. Animals were first sedated with intramuscular ketamine hydrochloride (20 mg/kg) followed by sodium pentobarbital (100 mg/kg) intravenously to achieve euthanasia.

### Vaccination and SIV-specific CD8+ T cell isolation

All 5 Mamu-A*01 positive rhesus macaques were vaccinated intravenously with 20 ng of SIVΔnef virus (supplied by Dr. Ronald Desrosiers) as described previously [14, 15]. APC-conjugated Mamu-A*01 MHC class I tetramers complexed with the cognate SIV Gag_181–189_ epitope (SIV Gag CM9 tetramers) [18] was kindly provided by Nancy Wilson and David Watkins (Wisconsin National Primate Research Center, Madison, WI). Peripheral blood samples were collected in EDTA tubes from vaccinated animals at week 3 (n = 5) and week 20 (n = 3) post-vaccination. After isolation, peripheral blood mononuclear cells (PBMCs) were stained with CM9 tetramer and anti-CD3 (SP34, FITC), anti-CD4 (L200, PerCP-Cy5.5) and anti-CD8 (RPA-T8, Alexa 700) antibodies. SIV Gag CM9 tetramer+ CD8+ T cells were sorted on a FACS Aria II cell sorter (BD Biosciences) as reported previously [17]. Over 5×10^3^ CM9-specific CD8+ T cells were yielded by sorting from each sample, with more than 99% purity.

### Sample preparation and microarray hybridization

Total RNAs were extracted from sorted cells using TRIzol (Life Technologies), followed by cDNA synthesis using Ovation Pico WTA System (Cat# 3300–12, NuGEN). After purification with QIAquick PCR Purification Kit (Cat# 28104, QIAGEN), the cDNA samples were labeled with biotin using Encore Biotin Module (Cat# 4200–12, NuGEN). All samples were assayed on GeneChip Rhesus Macaque Genome Array (Cat# 900656, Affymetrix Inc.) in Genomics Core Research Facility at University of Nebraska-Lincoln (UNL). The output files of microarray were deposited to the Gene Expression Omnibus (GEO) database under accession number GSE75567.

### Microarray data analysis

The data in CEL files from microarray assays were normalized using Robust Multi-array Analysis (RMA) correction algorithm, and Limma package [20] was then used for differential expression analysis. Genes with fold change larger than 2 and P < 0.01 were defined as differentially expressed genes (DEGs), which were further analyzed by ClueGO package of Cytoscape software [21] and literature search for gene function categories and pathway analysis.

## Results

### Different gene expression profiles of SIV Gag-specific CD8+ T cells at 20 versus 3 WPV

CD8+ T cells are thought to be critical for protecting against pathogenic SIV infection [22, 23]. However SIV-specific CD8+T cells elicited by SIVΔnef LAV are not quantitatively different from CD8+ T cells of other vaccination strategies [24], and SIV-specific CD8+ T cells quantitatively and temporally did not correlate with maturation of immune protection [15], suggesting that quality, rather than quantity, of SIV-specific CD8+ T cells may contribute to the time-dependent protection induced by SIVΔnef vaccine [15]. Epitope mapping study showed that, in rhesus macaques with Mamu-A*01, Gag CM9 epitope (sequence: CTPYDINQM) of cytotoxic T lymphocytes (CTL) is immunodominant and plays an important role in controlling SIV replication [18, 19].

**Fig 1:**
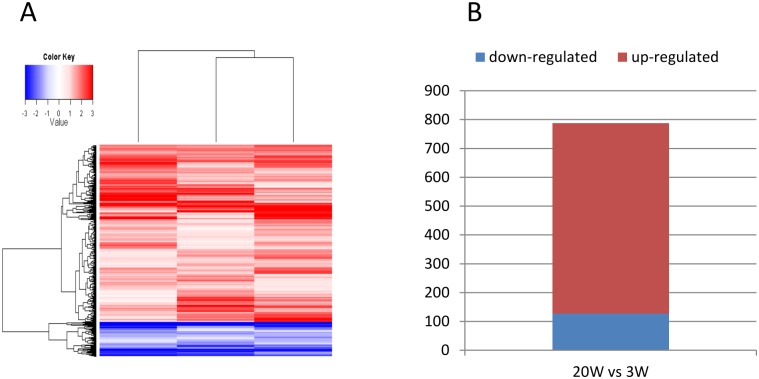
Vaccination induced qualitative differences at week 20 compared with week 3. (A) Heat map of gene expression at 20 WPV compared with 3 WPV, shown as log2 (fold changes). Up-regulated and down-regulated genes are color-coded as red and blue, respectively. (B). Histogram of number of up- and down-regulated genes at 20 WPV compared with 3 WPV.

To elucidate whether there are qualitative differences of gene expression in Gag-specific CD8+ T cells at 3 and 20 WPV and whether these differences, if validated, temporally correlate with emergence of protection, genome-wide transcriptional profiles were studied using rhesus microarray. As shown in Fig 1A, there were differentially expressed genes (DEGs) at 20 WPV as compared with 3 WPV. Of the total 788 DEGs at 20 WPV, 661 DEGs were up-regulated (Fig 1B, S1 Table), demonstrating clear differences in gene expression in Gag-specific CD8+ T cells from 3 to 20 WPV.

**Fig 2:**
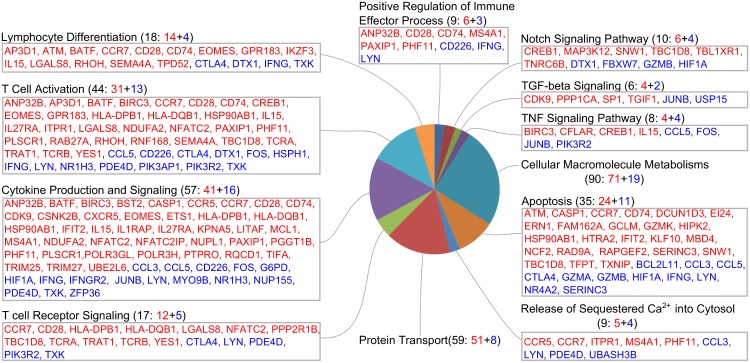
Categories of differentially expressed genes. Up-regulated and down-regulated genes are colored as red and blue, respectively.

To gain insights into the functions of these DEGs, gene function analysis was performed using ClueGO [21] and literature search. As shown in Fig 2, in the context of CD8+ T cells, the DEGs are involved in multiple functional categories, such as T cell function and differentiation, cytokine production and signaling, immune effector process, apoptosis, and macromolecule metabolism.

### Gag-specific CD8+ T cells at 20 WPV increased stimulatory/inhibitory molecule ratio

**Fig 3:**
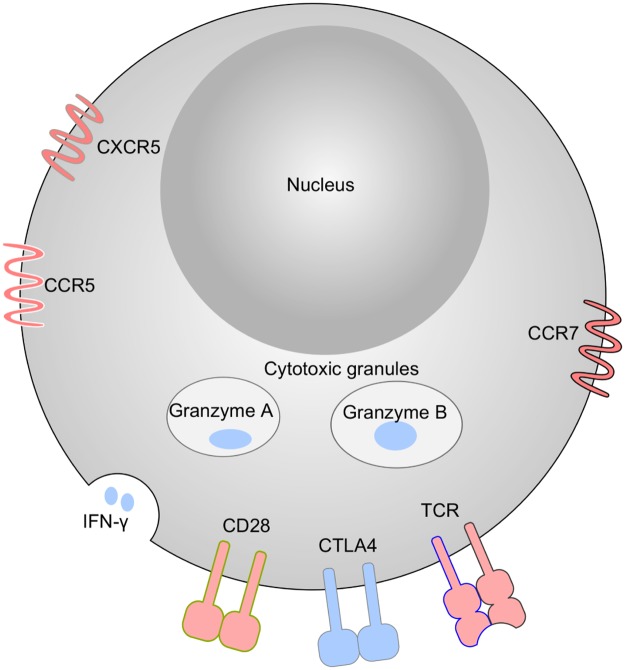
Schematic representative of phenotypes of Gag-specific CD8+ T cells at 20 WPV. Up-regulated and down-regulated genes are colored as red and blue, respectively.

As shown in Fig 2, DEGs are involved in T cell receptor signaling and T cell differentiation. CD8+ T cells are primarily signaled through T cell receptor α (TCRA) and β (TCRB), by MHC class I and peptide complex and through co-stimulatory molecule CD28 by CD80 (B7-1) and CD86 (B7-2). CTLA-4, a CD28 competitor on T cell surfaces with higher binding affinity to CD80 and CD86, serves as inhibitory molecule. As shown in Figs 2 and 3, the TCRA (3.5-fold), TCRB (6.4-fold) and CD28 (5.3-fold) were up-regulated at week 20 WPV, while CTLA-4 had 4.3-fold down-regulation. This pattern suggests that the Gag-specific CD8+ T cells were more functional at 20 WPV compared with 3 WPV. Down-regulation of Socs3, a protein that facilitates T cell exhaustion, also suggested improved T-cell functionality at 20 WPV.

Besides the up-regulation of stimulatory gene expression and down-regulation of inhibitory gene expression, the Gag-specific CD8+ T cells at 20 WPV also up-regulated MHC class II genes HLA-DPB1 (5.6-fold), HLA-DQB1 (3.6-fold), and CD74 (2.7-fold).

### Gag-specific CD8+ T cells at 20 WPV down-regulated effector molecule expression, and showed central memory properties as compared with 3 WPV

The Gag-specific CD8+ T cells at 20 WPV displayed an overall down-regulation of genes involved in effector functions as compared with 3 WPV. As shown in Fig 2, the Gag-specific CD8+ T cells at 20 WPV had lower expression of interferon-γ (IFNG, -5.3-fold), granzyme A (GZMA, -3.2-fold), granzyme B (GZMB, -4.3-fold), and CCL5/RANTES (-2.9-fold), but had elevated central memory functions, manifested by up-regulated expression of EOMES, IL-15, CCR7, BATF, and down-regulated expression of RORA, which regulates CD8+ T cell activation and effector functions (Figs 2 and 3 and S1 Table). Memory CD8+ T cells express higher level of EOMES [25], which is essential for long-term memory formation and homeostatic renewal [26]. Up-regulated IL-15 may also help maintain longevity of memory CD8+ T cells [27], and increased CCR7 expression may help CD8+ home to T cell zone in the secondary lymphoid tissues. Furthermore, it was reported that while naive and antigen-specific effector memory CD8+ T cells have CCR5+CCR7- and CCR5-CCR7+ phenotypes, antigen-specific and less differentiated memory CD8+ T cells have CCR5+CCR7+ phenotype[28, 29]. The Gag-specific CD8+ T cells at 20 WPV upregulated both CCR7 (5.6-fold) and CCR5 (2.4-fold). Of note, Gag-specific CD8+ T cells at 20 WPV also upregulated CXCR5 (3.9-fold), which may help some of Gag-specific CD8+ cells home to B follicles in the secondary lymphoid tissues.

## Discussion

The Nef protein plays important roles in HIV-1/SIV pathogenesis *in vivo*, although it is not required for viral replication *in vitro*[30]. Individuals infected with HIV-1 with defective *nef* are associated with slow disease progression [31], and the pathogenicity of SIV after deleting of *nef* is significantly attenuated *in vivo* in rhesus macaques. SIVΔnef live attenuated vaccine (LAV) induces potent protection against subsequent intravenous challenge with pathogenic wild-type SIVmac251 in a time-dependent manner from limited protection at 5 WPV to potent protection at 15 WPV and thereafter [6, 17]. Previous studies demonstrated that SIV-specific CD8+ T cells elicited by SIVΔnef vaccine contribute to the time-dependent protection [2, 9–12]. Recently we found that IgG antibodies against SIV gp41 trimers correlated spatially and temporally with the maturation of local protection against high-dose pathogenic SIV vaginal challenge [14]. However, the quantity and location of Gag-specific CD8+ T cells in female genital and lymphoid tissues induced by SIVΔnef vaccine do not correlate with protection against vaginal challenge [15]. Billingsley and colleagues found that the transcriptional factor profiles of SIV-specific CD8+ T cells after SIVΔnef vaccine changed over time and temporally associated with protection, indicating SIV-specific CD8+ T cells elicited by SIVΔnef may be qualitatively different between time points at which protection is and is not observed [17].

To further characterize the Gag-specific CD8+ T cells at the time points of protection and no/limited protection, in this study, the global gene expression patterns of Gag-specific CD8+ T cells at 3 and 20 WPV were compared. Our data showed that Gag-specific CD8+ T cells at 20 WPV have more central memory-like properties: higher expression of CCR7, EOMES, IL-15, and lower effector gene expression as discussed above. After infection, antigen-specific CD8+ T cells undergo clonal expansion and differentiate into effector memory T cells that control the infection, while a small percentage of antigen-specific CD8+ T cells differentiate into central memory T cells with lower killing ability but higher proliferation capacity [32]. After encountering the original pathogen, central memory CD8+ T cells mount stronger and quicker responses, thus central memory CD8+ T cells can greatly contribute to recall responses to control infection [33]. Our results are consistent with the results of a recent study of SIVΔnef-induced Gag-specific CD8+ T cells [17] and another study showing that most of HIV-specific CD8+ T cells from elite controllers (EC) have central memory phenotype [34]. Central memory CD8+ T cells, although existing at low frequencies in EC, have strong proliferative capacity and efficiently control HIV-1 replication [34]. The up-regulated CCR7 expression in central memory CD8+ T cells facilitates homing to secondary lymphoid tissues, where, upon exposure to antigen again, they can be stimulated to proliferate and differentiate for virus control. In addition, our results showed that the Gag-specific CD8+ T cells also up-regulated chemokine receptor CXCR5 (3.9-fold). Recent studies indicated that antigen-specific CD8+ T cells that express CXCR5 can migrate into B cell follicles of the secondary lymphoid tissues [35–37]. Therefore it is plausible to speculate that the up-regulated expression of CXCR5 in Gag-specific CD8+ T cells may reflect a subset of Gag-specific CD8+ T cells induced by the SIVΔnef vaccine can home to B cell follicles.

Our results also show that, compared with week 3, Gag-specific CD8+ T cells at week 20 are more functional, with up-regulated genes involved in T cell receptor signaling (TRA and TRB) and co-stimulation (CD28). CD28-B7 interaction is necessary for generating class I major histocompatibility complex-specific CTL[35]. It has been demonstrated that lack of CD28 expression on HIV-specific CD8+ T cells is associated with fast disease progression [36, 37]. CTLA-4 is a key negative regulator for T cell function and exerts inhibitory effects by competing with CD28 for ligands CD80 and CD86. It was reported that CTLA-4 blockade induces tumor regression in several murine model [38, 39] and in patients with metastatic melanoma in clinical trial [40, 41]. CTLA-4 blockade was also associated with decreased SIV RNA levels in lymph nodes and an increase in the effector function of SIV-specific CD8+ T cells in rhesus macaques [42]. The up-regulation of CD28 (5.3 fold) and down-regulation of CTLA-4 (-4.3 fold) suggest that Gag-specific CD8+ T cells at 20 WPV are more functional. In addition, compared with CD8+ T cells from 3 WPV, the Gag-specific CD8+ T cells at 20 WPV also showed lower levels of granzyme A and B expression. Delivery of granzyme A and B to target cells results in an efficient elimination of CD4+ T cells infected with HIV-1 [43]. Because of lower titer of SIVΔnef virus and less infected cells in vaccinated macaques at 20 WPV than 3 WPV, it is plausible that the effector molecule granzyme A and B had lower expression. Consistent with our observation, a study in influenza virus showed that, compared with acute infection, GZMA and GZMB mRNA level decreased more than 2-fold in memory CD8+ T cells [44]. Interestingly, granzyme K (GZMK) was up-regulated at 20 WPV. Granzyme K expresses at much lower level than granzyme A, but with higher specificity [45]. GZMA and GZMB deficient mice can still launch effective and rapid killing of target cells, and the retained cytotoxicity may because expression of GZMK [46]. And the roles of up-regulated GZMK in these Gag-specific CD8+ T cells induced by SIVΔnef vaccine need further investigation.

Overall, this study shows that Gag-specific CD8+ T cells at week 20 are qualitatively different from cells at week 3, and have properties of central memory T cells. The high level expression of CCR7, CXCR5 and other chemokine receptors in SIV-specific CD8+ T cells may direct these cells trafficking to lymphoid tissues, and even to B follicles. And up-regulated expression of genes involved in T cell signaling, especially up-regulation of CD28 and down-regulation of CTLA-4, may render these cells functionally more capable, resulting in efficient control of subsequent pathogenic SIV infection by quickly eliminating infected cells. Collectively, our study indicates that a higher quality of SIV-specific CD8+ T cells elicited by SIVΔnef LAV over time contributes to the maturation of time-dependent protection.

## Supporting information

S1 Table List of differentially expressed genes at 20 WPV compared with 3 WPV. (XLSX)
